# Adjuvant transcranial direct current stimulation for treating Alzheimer's disease: A case study

**DOI:** 10.1590/S1980-5764-2016DN1002013

**Published:** 2016

**Authors:** Suellen Marinho Andrade, Camila Teresa Ponce Leon de Mendonça, Thobias Cavalcanti Laurindo Pereira, Bernardino Fernandez-Calvo, Regina Coely Neves Araújo, Nelson Torro Alves

**Affiliations:** 1 Cognitive Neuroscience and Behavior Program. Department of Psychology, Universidade Federal da Paraíba, João Pessoa PB, Brazil.

**Keywords:** electrical stimulation, dementia, Alzheimer's disease, cognitive therapy

## Abstract

We report the case of a 73-year-old male patient with Alzheimer's disease who underwent 10-daily transcranial direct current stimulation (tDCS) sessions. tDCS was applied over the left dorsolateral prefrontal cortex as an adjuvant to the traditional treatment that the patient was receiving, which consisted of anticholinergic medication and cognitive training. The data were qualitatively analyzed and are presented in an analytic and structured form. The effects on cognitive performance were evaluated using the Alzheimer's Disease Assessment Scale-cognitive subscale as the primary outcome. Secondary outcomes were assessed with a set of tests consisting of the Neuropsychiatric Inventory, the Blessed Dementia Scale and the Disability Assessment for Dementia. The data obtained revealed that the application of tDCS had a stabilizing effect on overall patient cognitive function and led to improved performance on all the secondary outcome tests. These preliminary results indicate that tDCS is a potential adjuvant therapeutic tool for cognitive rehabilitation in Alzheimer's disease .

## RESUMO

Nós relatamos o caso de um paciente com 73 anos de idade, diagnosticado com doença de Alzheimer, submetido a 10 sessões diárias de estimulação transcraniana por corrente continua (ETCC). A ETCC foi aplicada sobre o córtex dorsolateral esquerdo como terapêutica adjuvante ao tratamento tradicional que o paciente recebia (medicação anticolinérgica e treino cognitivo). Os dados foram analisados qualitativamente e apresentados em um formato analítico e estruturado. O desempenho foi avaliado através da Alzheimer's Disease Assessment Scale-cognitive subscale, como desfecho primário. Os desfechos secundários incluíram: Neuropsychiatric Inventory, Blessed Dementia Scale e Disability Assessment for Dementia. Os dados obtidos demonstraram que a ETCC teve um efeito estabilizador sobre a função cognitiva geral do paciente e levou ao aumento do desempenho em todos os testes de desfechos secundários. Estes resultados preliminares indicam que a ETCC é uma potencial terapêutica adjuvante para a reabilitação cognitiva na doença de Alzheimer.

## INTRODUCTION

Alzheimer's disease (AD) is the most common form of dementia in the elderly population and is characterized by cognitive and behavioral changes that interfere with social relationships and functional activities.^1^

Non-pharmacological strategies have been used to delay these cognitive deficits and minimize functional impairment. One of these strategies is transcranial direct current stimulation (tDCS), a low-cost, portable, safe method that is capable of modulating cortical activity and inducing neuroplasticity mechanisms.^2^ However, there is a dearth of studies evaluating cognitive and behavioral measures following treatment for AD with tDCS, especially when combined with other traditional treatments, such as anticholinergic medication or cognitive therapy.^3^

This case report provides evidence of the efficacy of tDCS as an adjuvant therapy in the treatment of patients with Alzheimer's; this therapy was associated with improved performance on cognitive and behavioral tests.

## METHODS

Case history. A 73-year-old male patient with higher education was referred to our research center by the National Alzheimer's Association. Two years prior, the patient had been diagnosed with AD according to the criteria of the National Institute of Neurological and Communicative Disorders and Stroke - Alzheimer's Disease and Related Disorders Association (NINCDS-ADRDA). The patient had mild cognitive impairment and a score of 1 on the Clinical Dementia Rating scale.^4^

The patient's family reported several AD symptoms, including difficulty remembering recent events, language impairment, spatiotemporal disorientation and decreased decision-making capacity; these symptoms interfered with his independence in activities of daily living (ADL). 

The patient was receiving treatment with donepezil (10 mg/day for 12 months) and sertraline (50 mg/day for 6 months) for symptoms of depression that had arisen after the AD diagnosis and were currently in remission (a score of 6 according to the Cornell Scale for Depression in Dementia). In addition to medication, the patient was undergoing cognitive training sessions with a neuropsychologist twice per week. These sessions involved tasks related to memory, language, attention, executive functions and praxis.

The local Ethics Committee approved the study protocol (44388015.7.0000.5188), and the patient signed an informed consent form agreeing to participate in the study. Medication use and cognitive therapy sessions continued throughout the study, with tDCS used as an adjuvant treatment for AD. 

Measures. Assessments were performed at two different time points, one week prior to (T0), and one week after (T1), the neurostimulation protocol. The ADAS-Cog was used for the primary outcome assessment. This scale includes items related to memory, language, praxis and orientation. To measure secondary outcomes, the following instruments were applied: the Neuropsychiatric Inventory (NPI),^5^ for the evaluation of non-cognitive AD symptoms, such as delusions, hallucinations, euphoria and apathy; the Blessed Dementia Scale,^6^ which assesses ADL, changes in habits and behavior disorders; and the Disability Assessment for Dementia (DAD),^7^ which quantitatively measures functional abilities in ADL and cognitive dimensions of impairments in ADL in relation to executive functions. 

A questionnaire on adverse effects was administered to assess the safety of the method. The patient was asked at each stimulation session whether he experienced any discomfort, which was graded on a Likert scale from 0 to 5 points for intensity of the experience.^8^

tDCS. tDCS was applied with a 2 mA current for 30 minutes on 10 consecutive days (excluding weekends). The TCT neurostimulator (Research version; Trans Cranial Technologies) was used, with 5 x 7 cm (35 cm^2^) electrodes wrapped in sponges moistened with saline (NaCl 0.9%). The anode was placed over the dorsolateral prefrontal cortex (region F3 according to the international 10-20 electroencephalography [EEG] system).^9^ The reference electrode was placed over the supraorbital region.

## RESULTS

The data were qualitatively analyzed and are presented in an analytic and structured form. Table 1 shows the changes in scores and percentage improvement with treatment.^10^ At baseline, the patient had significant deficits in recalling words (ADAS-Cog subscale) and in execution efficacy (DAD subscale). For the tests applied, negative values indicate an improvement after treatment (T1) because higher scores on these scales denote more severe cognitive impairment. Specifically, the patient's improvement was greater on the domains that were most affected at baseline, i.e., those related to verbal memory and executive functions. Clinically, the patient's family reported improvements in decision-making and ADL. Regarding adverse events, the therapy was well tolerated; the only discomfort reported was temporary itching during the initial minutes of neurostimulation. 

**Table 1:** Cognitive performances of patient before and after tDCS application

Neuropsychological tests	T0	T1	Change scores*	%improvement**
ADAS-Cog				
Word Recall Test	6	5	-1^#^	16.0
Naming objects and Fingers	0	0	0	0
Commands	1	0	-1^#^	100
Constructional Praxis	1	1	0	0
Ideational Praxis	2	2	0	0
Orientation	2	1	-1^#^	50.0
Word Recognition Task	2	2	0	0
Remembering Test Instructions	1	1	0	0
Spoken Language Ability	1	0	-1^#^	100
Word Finding Difficulty	0	0	0	0
Comprehension	0	0	0	0
Total	16	12	-4^#^	25.0
NPI-Q	42	14	-28^#^	66.6
BDS	7	4,5	-2.5^#^	35.7
DAD				
DAD-ADL	94.1	100	5.9	6.2
DAD-IADL	83.0	83.0	0	0
Leisure	66.6	100	33.3	50.0
Initiation	90.9	90.9	0	0
Planning and organization	87.5	87.5	0	0
Effective performance	87.5	93.7	6.2	7.1
Total	88.5	91.4	2.9	3.2

ADAS-Cog: Alzheimer's Disease Assessment Scale-Cognitive Subscale; NPI-Q: Neuropsychiatric Inventory Questionnaire; BDS: Blessed Dementia Scale; DAD: Disability Assessment for Dementia. *Change scores [difference between post (T1) and pre-intervention (T0)]. **Percentages improvement from baseline were calculated with the equation: [(T1-T0)/T0] x 100. ^#^Negative values on the ADAS-Cog, NPI-Q and BDS categories in Table 1 indicate improvement.

## DISCUSSION

It is well established in the literature that anticholinergic medication and cognitive therapy can provide AD patients with some benefits.^11,12^ The present study has expanded research on AD treatments by showing that tDCS is capable of producing cognitive and behavioral changes. We observed that this method was safe and had minimal adverse effects. 

The patient in our case study presented a relevant decrease in ADAS-Cog scores equivalent to the improvement achieved by patients who undergo several months of traditional treatment. A systematic review of studies evaluating the effects of medication on AD found that during the initial 6-12 months, patients treated with cholinesterase inhibitors had an average improvement of 2.7 points.^13^ However, a study of 784 patients treated with donepezil, rivastigmine or galantamine found that 49% of patients had improved or unchanged ADL and that 51% experienced a decrease in independent living.^14^

In this context, our recommendation for the use of tDCS would not be to replace drug or cognitive treatment for AD patients. Instead, tDCS would function in a complimentary manner as an adjuvant, enhancing gains obtained or preventing rapid disease progression, as previously observed in studies involving neuromodulation and behavior therapy in AD patients.^15,16^ A double-blind, placebo-controlled, randomized trial with 15 AD patients found that transcranial magnetic stimulation, a method similar to tDCS, combined with cognitive therapy, was able to reduce the average ADAS-Cog score by 3.76 points in the treated group after 6 weeks compared to the placebo group.^17^ A recent study reported that the effects of tDCS on a patient's overall cognitive function persisted for up to 3 months of follow-up, supporting the use of tDCS as an adjuvant tool in AD treatment.^18^

Although there is evidence of improvements in behavior and function in AD patients undergoing tDCS treatment,^2^ ^,^ ^19^ the mechanisms underlying these effects on cognitive functions are not fully understood; the effects might be related to neural plasticity and the actions of different neurotransmitters.^20^ tDCS has been used to increase cortical activity in AD patients because this patient group exhibits temporoparietal hypoactivity.^21^ The literature shows that tDCS alters neural activity and blood flow in the brain, causes synaptic and non-synaptic after-effects, and can modify neuronal polarization and functional connectivity patterns in the brain.^22^

In the present case, we were not able to evaluate changes in cortical or subcortical activity because our measurements were not based on neuroimaging or biomarker methods. Additional studies using these methodological approaches are needed to clarify the relationship between the observed improvements on cognitive tests and changes at the cellular level.

Our results are in agreement with previously published studies reporting positive changes in behavior after tDCS. These findings are promising and indicate that tDCS is an option for adjuvant treatment to improve the prognosis of AD patients.
